# Etymologia: Chagas Disease

**DOI:** 10.3201/eid1910.ET1910

**Published:** 2013-10

**Keywords:** etymologia, Chagas disease, Carlos Chagas, Oswaldo Cruz, Trypanososma cruzi, parasites, triatomine insects, cardiac disease, gastrointestinal disease

## Cha·gas [shä-gəs] Disease

Prevalent among persons who have lived in Mexico, Central America, and South America, Chagas disease can cause chronic and potentially severe cardiac and gastrointestinal disease decades after infection. The disease is named for Carlos Chagas, a Brazilian scientist who discovered a new species of *Trypanosoma* in the intestines of triatomine insects (called *barbeiro *or barber because they often bite the face). In 1908, Chagas named the new species *T. cruzi* after his mentor, Oswaldo Cruz. The next year, he identified the parasite in the blood of an ill 2-year-old girl named Bérénice, in what became the first description of this new human disease.
